# Enhancing Therapeutic Approaches in Glioblastoma with Pro-Oxidant Treatments and Synergistic Combinations: In Vitro Experience of Doxorubicin and Photodynamic Therapy

**DOI:** 10.3390/ijms25147525

**Published:** 2024-07-09

**Authors:** Bruno Agustín Cesca, Matías Daniel Caverzan, María Julia Lamberti, Luis Exequiel Ibarra

**Affiliations:** 1 Departamento de Biología Molecular, Facultad de Ciencias Exactas, Fisicoquímicas y Naturales, Universidad Nacional de Rio Cuarto, Rio Cuarto X5800BIA, Argentina; brunocesca22@gmail.com (B.A.C.); mlamberti@exa.unrc.edu.ar (M.J.L.); 2 Departamento de Patología Animal, Facultad de Agronomía y Veterinaria, Universidad Nacional de Rio Cuarto, Rio Cuarto X5800BIA, Argentina; dcaverzan@ayv.unrc.edu.ar; 3 Instituto de Investigaciones en Tecnologías Energéticas y Materiales Avanzados (IITEMA), Universidad Nacional de Rio Cuarto (UNRC), Consejo Nacional de Investigaciones Científicas y Técnicas (CONICET), Rio Cuarto X5800BIA, Argentina; 4 Instituto de Biotecnología Ambiental y Salud (INBIAS), Universidad Nacional de Rio Cuarto (UNRC), Consejo Nacional de Investigaciones Científicas y Técnicas (CONICET), Rio Cuarto X5800BIA, Argentina

**Keywords:** photodynamic therapy, chemotherapy, glioblastoma, adjuvant treatment, oxidative therapy, TP53 mutation, PTEN mutation

## Abstract

Glioblastoma (GBM) is an aggressive brain cancer characterized by significant molecular and cellular heterogeneity, which complicates treatment efforts. Current standard therapies, including surgical resection, radiation, and temozolomide (TMZ) chemotherapy, often fail to achieve long-term remission due to tumor recurrence and resistance. A pro-oxidant environment is involved in glioma progression, with oxidative stress contributing to the genetic instability that leads to gliomagenesis. Evaluating pro-oxidant therapies in brain tumors is crucial due to their potential to selectively target and eradicate cancer cells by exploiting the elevated oxidative stress levels inherent in these malignant cells, thereby offering a novel and effective strategy for overcoming resistance to conventional therapies. This study investigates the therapeutic potential of doxorubicin (DOX) and photodynamic therapy (PDT) with Me-ALA, focusing on their effects on redox homeostasis. Basal ROS levels and antioxidant gene expression (NFE2L2, CAT, GSR) were quantitatively assessed across GBM cell lines, revealing significant variability probably linked to genetic differences. DOX and PDT treatments, both individually and in combination, were analyzed for their efficacy in inducing oxidative stress and cytotoxicity. An in silico analysis further explored the relationship between gene mutations and oxidative stress in GBM patients, providing insights into the molecular mechanisms underlying treatment responses. Our findings suggest that pro-oxidant therapies, such as DOX and PDT in combination, could selectively target GBM cells, highlighting a promising avenue for improving therapeutic outcomes in GBM.

## 1. Introduction

According to the World Health Organization (WHO), glioblastoma (GBM) is a particularly aggressive type of cancer that accounts for 48.6% of malignant tumors in the brain and 14.5% of all central nervous system (CNS) tumors [1]. The cellular origin for this Grade 4 glioma is still debated, highlighting its significant variability and cellular hierarchies, which mainly arise from stem or progenitor cells and that confer considerable intra- and intertumoral heterogeneity [2]. 

The current treatment for GBM often involves a step-by-step process starting with surgical removal of the tumor, followed by radiation and chemotherapy with temozolomide (TMZ). Nonetheless, the molecular diversity inherent in GBM and the formidable barrier presented by the blood–brain barrier (BBB) pose significant challenges in achieving the complete eradication of tumor cells. Poor prognostic outcomes in GBM patients often result from extensive intertumoral and intratumoral heterogeneity, as well as the occurrence of postoperative relapses and development of treatment resistance, phenomena observed in approximately half of the patients undergoing TMZ therapy [3]. Hence, it is critically important to explore alternative therapeutic avenues, as conventional treatments seldom result in a cure. 

Oxidative stress plays a crucial role in GBM cells, influencing both tumor progression and therapeutic response [4]. GBM cells typically exhibit elevated levels of reactive oxygen species (ROS) due to their high metabolic activity and inherent genetic instability [5,6]. Oxidative stress relies in large part on a mismatch between the cellular antioxidant defense system and the excess formation of ROS, including free radicals, which are molecules with unpaired electrons at the outer orbitals. This increased oxidative stress contributes to the aggressive nature of the tumor by promoting DNA damage, mutagenesis, and cellular proliferation. Additionally, the dysregulation of antioxidant defenses in GBM cells creates a vulnerability that can be therapeutically exploited [5,7]. Although initially appearing counterintuitive due to the association of ROS with cellular damage and malignant transformation, the selective induction of oxidative stress within cancer cells holds therapeutic promise, particularly for aggressive malignancies such as GBM [4,5]. By further increasing ROS levels through pro-oxidant therapies, it is possible to push the oxidative stress beyond the threshold that cancer cells can tolerate, leading to their selective eradication while sparing normal cells. Therefore, understanding the dynamics of oxidative stress in GBM cells is essential for developing effective treatments and improving patient outcomes. Furthermore, the elevated ROS levels induced by pro-oxidant therapies can surpass the oxidative stress threshold in cancer cells, leading to apoptosis and subsequent elimination of malignant cells [8]. 

Recent trends in preclinical [3,9] and clinical studies [10] of GBM have focused on the use of doxorubicin (DOX), one of the earliest chemotherapy agents (CTA) in cancer treatment. Doxorubicin is considered a pro-oxidant therapy due to its ability to generate reactive oxygen species (ROS) within cancer cells. This CTA induces oxidative stress through various mechanisms. It can undergo redox cycling in the presence of cellular reductases, resulting in the production of superoxide anions and other ROS. Additionally, it can also enhance the Fenton reaction by chelating iron and interfering with the electron transport chain in the mitochondria, leading to increased ROS production [11,12]. Conversely, Photodynamic Therapy (PDT) has emerged as a promising alternative therapeutic modality for GBM, due to its ability to selectively target cancerous cells while minimizing damage to surrounding healthy tissue by generating oxidative stress [13]. Ongoing clinical trials aim to further elucidate the efficacy of PDT in GBM treatment [14,15]. An advantage of using photosensitizers (PS) in PDT for GBM is their ability to function as photodiagnostic agents for evaluating the presence of any residual cells after surgery [16]. Moreover, PDT exhibits the capability to synergize with other treatment modalities within a combination framework [17]. These combination therapies often entail the administration of multiple anticancer agents in reduced dosages compared to individual administration, thereby enhancing efficacy, reducing toxicity, and mitigating the development of drug resistance by targeting diverse cancer pathways.

Key mutations found in GBM patients, including the amplification of the epidermal growth factor receptor (EGFR), as well as mutations in the genes encoding phosphatase and tensin homolog (PTEN) and tumor protein 53 (TP53), have been linked to the response to treatments and the accumulation of ROS, which play a significant role in gain-of-function (GOF) activities in cancer cells [18,19]. Accumulating evidence underscores the pivotal role of these GOF activities in promoting tumor progression and conferring resistance to various anticancer therapies, thereby underscoring the imperative to comprehend their underlying mechanisms in the context of therapeutic interventions [20]. Moreover, growing evidence suggests that cells harboring mutations in *TP53* may exhibit heightened susceptibility to pro-oxidant drugs compared to those with the wild-type form of this gene [21,22,23,24]. This heightened sensitivity facilitates the accumulation of deleterious ROS, ultimately leading to cellular damage and the demise of cancer cells.

To evaluate the impact of pro-oxidant treatments, such as DOX chemotherapy and PDT with Me-ALA contemplating GBM heterogeneity, we propose examining their effects on various human GBM cell lines with different mutation conditions. Our study will encompass both individual treatment approaches and combined therapeutic regimens. Furthermore, an in silico study was conducted to investigate the correlation between specific gene mutations and the abnormal regulation of oxidative stress in GBM patients. This investigation aimed to provide an explanation for the observed behavior in various cell line models.

## 2. Results 

### 2.1. The Impact of Pro-Oxidant Therapies on the Cellular Viability of GBM Cells as Monotherapies

Oxidative stress in glioma, caused by high levels of ROS leads to more aggressive tumors. The weakness in antioxidant defenses in GBM cells can be therapeutically exploited. Evaluating pro-oxidant treatments, such as DOX and PDT, that increase ROS levels can offer new insights into potential therapeutic approaches, overcoming resistance to conventional therapies. 

**Figure 1:**
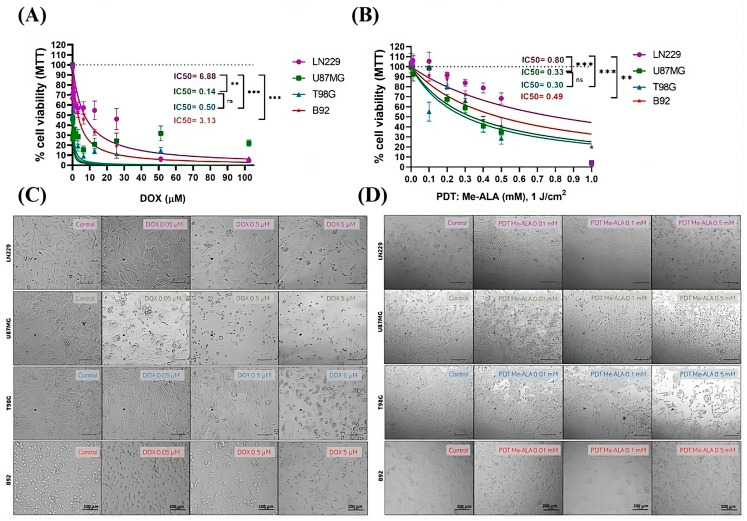
Cell viability after DOX and PDT treatments as monotherapies. Quantification of cell viability by MTT assay 24 h after DOX (**A**) or PDT (**B**) treatments for U87MG, T98G, LN229 and B92 cell lines as a function of DOX concentration and Me-ALA concentration and a light dose of 1 J/cm^2^, respectively. Cell viability percentages were normalized to control cells not exposed to treatments. Statistical analysis was performed using one-way ANOVA with Tukey’s post-hoc test. Significance levels are denoted by asterisks ** *p* < 0.01, *** *p* < 0.001 and ns: no statistically significant differences. Alterations in morphology following treatment with varying doses of DOX (0, 0.05, 0.5 and 5 μM) over 24 h in GBM cell lines and glial B92 cells (**C**) and after PDT with Me-ALA at different concentrations (0, 0.01, 0.1 and 0.5 mM) (**D**). The cell lines treated were captured using an inverted optical microscope, which produced bright-field images. Scale bars = 100 and 200 μm.

To assess DOX, GBM cells (LN229, U87MG, and T98G) and rat glial cells (B92) were treated with varying concentrations of DOX for 24 h. A clear trend emerged with U87MG showing the highest sensitivity (IC50 of 0.14 ± 0.1 µM) and LN229 exhibiting the greatest resistance (IC50 of 6.88 ± 0.6 µM). T98G cells had an intermediate response (IC50 of 0.5 ± 0.15 µM) (Figure 1A). On the other hand, B92 glial cells showed a less pronounced susceptibility to DOX with an IC50 of 3.13 ± 0.3 µM. MTT results were supported by observation of cellular morphological changes consistent with cell death. Upon comparing the effects induced by the use of CTA, lesser morphological changes are observed for the LN229 and B92 cell lines in comparison to U87MG and T98G at equivalent concentrations of DOX (Figure 1C).

The effects of PDT with different Me-ALA concentrations were also evaluated. Me-ALA functions as a prodrug, undergoing bioconversion to the endogenous photosensitizer PpIX [25]. Both Me-ALA in concentrations up to 2 mM and irradiation alone with light doses up to 20 J/cm^2^ did not induce cell death alone (Figure S1). Our study found that the LN229 cell line exhibited the highest level of resistance to PDT with Me-ALA, whereas the T98G and U87MG cell lines were the most susceptible (Figure 1B). The IC50 values for PDT were 0.8 ± 0.2 mM; 0.33 ± 0.05 mM; 0.3 ± 0.1 mM and 0.49 ± 0.07 for LN229, U87MG, T98G and B92, respectively. Results were supported by morphological changes observed after PDT treatment (Figure 1D). PDT with Me-ALA was less effective in achieving cell death in B92 glial cells, which can be attributed to their lower capacity for PpIX formation compared to GBM cell lines (Figure S2).

**Figure 2:**
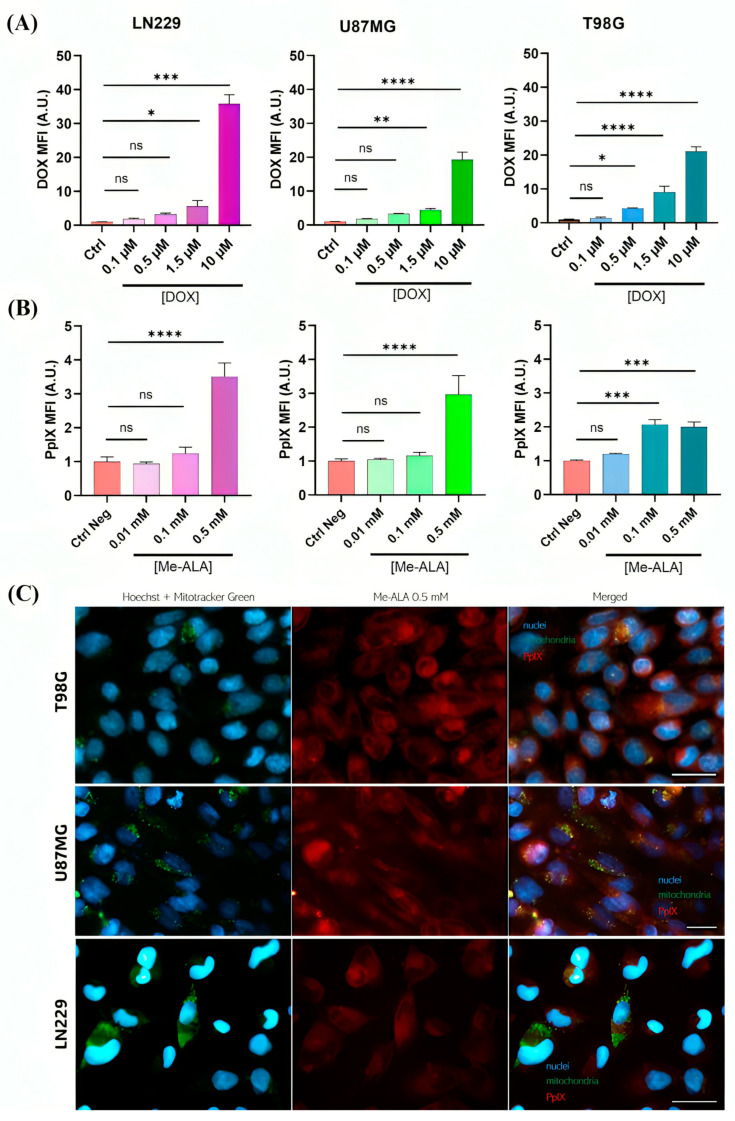
DOX incorporation and PpIX bioproduction. (**A**) DOX uptake evaluation by flow cytometry in GBM cell lines after 24 h of incubation with different DOX concentrations. (**B**) PpIX bioproduction evaluation by flow cytometry in GBM cell lines after 4 h of incubation time. Both DOX uptake and PpIX intracellular levels were represented by fluorescence intensity fold increments (MFI, arbitrary units A.U. compared with control cells) in the red channel of FC. Statistical analysis was performed using one-way ANOVA with Tukey’s post-hoc test. Significance levels are denoted by asterisks * *p* < 0.05, ** *p* < 0.01, *** *p* < 0.001 and **** *p* < 0.0001, ns: no statistically significant differences. (**C**) Fluorescence microscopy images of stained GBM cells. The images show the nuclei stained with Hoechst (blue), mitochondria stained with MitoTracker (green), and PpIX production visualized (red). Cells were incubated with Me-ALA to induce PpIX formation. Scale bar = 50 µm.

Considering that pro-oxidant treatments must generate intracellular ROS, which depends on the internalization of both DOX and the prodrug Me-ALA, both processes were evaluated using fluorescence microscopy and flow cytometry (Figure S3). Fluorescence emission of DOX and PpIX subsequent to the incubation with Me-ALA, was analyzed by flow cytometry to quantify the cellular uptake of DOX and the production of PpIX. An increase in DOX concentration resulted in a corresponding rise in cellular uptake (Figure 2A). Additionally, a concentration-dependent increase in PpIX production was observed in each of the three GBM cell lines with increasing Me-ALA concentrations (Figure 2B). Furthermore, PpIX was identified in GBM tumor cells, displaying partial co-localization with mitochondria and also exhibiting fluorescence in the cytoplasm, which is attributed to the incubation time and subsequent formation and accumulation of PpIX (Figure 2C). In fact, the conversion of ALA to PpIX involves eight enzymatic steps, with four occurring in the mitochondria and the remaining four in the cytosol [26]. Furthermore, the intracellular localization of PpIX, which was produced by incubating with Me-ALA, was assessed by colocalization studies employing the WGA probe. The WGA probe specifically stains the glycoproteins and glycolipids present on the cell membrane (Figure S2).

### 2.2. The Impact of Pro-Oxidant Therapies on ROS Production in GBM Cells as Monotherapies

**Figure 3:**
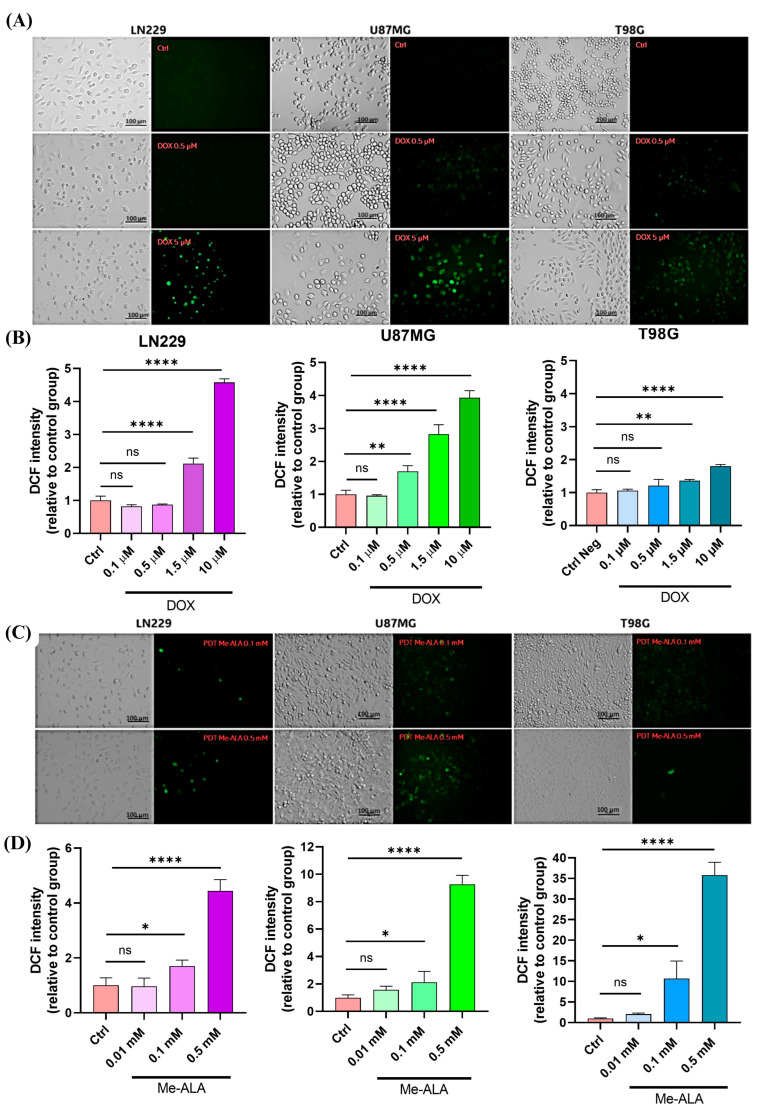
ROS production evaluation after monotherapies. Geometric mean fluorescence intensity quantification relative to autofluorescence of control group for LN229, U87MG and T98G after DOX treatment (**A**) and PDT with Me-ALA (**C**). Fluorescence microscopy evaluation of intracellular ROS levels after DOX treatment (**B**) and PDT with Me-ALA (**D**). ROS levels were determined immediately after each treatment with DCFDA assay using flow cytometry and fluorescence microscopy, scale bar = 100 µm. Statistical analysis was performed using two-way ANOVA with Tukey’s post-hoc test. Significance levels are denoted by asterisks * *p* < 0.05, ** *p* < 0.01and **** *p* < 0.0001, ns: no statistically significant differences.

DOX treatment significantly increased ROS levels in all cell lines, with higher concentrations (1.5, 5 and 10 μM) inducing substantial oxidative stress. Notably, LN229 and U87MG cells exhibited greater increases in ROS compared to T98G (Figure 3A,B).

PDT with Me-ALA treatment significantly increased ROS production, more so than DOX at the tested concentrations (0.1 and 0.5 mM), highlighting the pro-oxidant nature of PDT. T98G and U87MG cell lines exhibited higher ROS levels with PDT compared to DOX (Figure 3C,D), with U87MG showing the highest oxidative stress, followed by T98G and LN229. This was consistent with PpIX bioproduction and cell viability results, showing a link between endogenous PS production and the cytotoxicity effects of ROS-mediated PDT. U87MG cells experienced the most stress and cytotoxic impact from PDT, whereas LN229 showed significant resistance with minimal oxidative stress. ROS production was additionally assayed by fluorescence microscopy. The fluorescence signal from DCF predominantly localized within the cytoplasmic regions of the tumor cells, suggesting active ROS generation in response to pro-oxidant treatments by itself and in combination (Figure 3A,C). On the other hand, B92 glial cells experienced less oxidative stress, as evidenced by the lower fluorescence emission of DCF compared to GBM cells (Figure S4). These results emphasize that both DOX and PDT efficiently induce oxidative stress in GBM cells, indicating their potential as treatments targeting tumor cells via ROS. However, the LN229 cell line, despite increased ROS levels, displays notable resistance to both treatments, suggesting a complex relationship between ROS production and cell viability.

### 2.3. N-Acetylcysteine (NAC) Rescues Pro-Oxidant Cytotoxicity Induced by DOX and PDT Therapies

**Figure 4:**
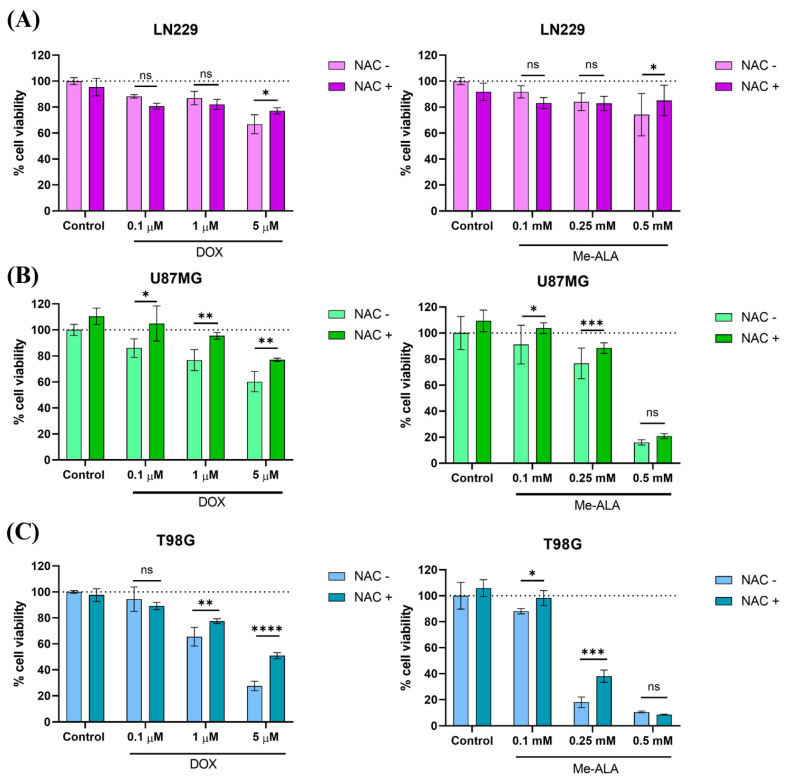
Evaluation of cytoprotective effects of NAC pretreatment against pro-oxidant treatments. Effect of protective effect of NAC against pro-oxidant-induced cell death in LN229 (**A**), U87MG (**B**) and T98G (**C**) GBM cell lines. Cells were incubated with 5 µM NAC for 24 h before exposure to DOX or PDT with Me-ALA. Cytotoxicity was determined with the MTT-based assay. Statistical analysis was performed using two-way ANOVA with Tukey’s post-hoc test. Significance levels are denoted by asterisks * *p* < 0.05, ** *p* < 0.01, *** *p* < 0.001 and **** *p* < 0.0001, ns: no statistically significant differences.

Cysteine, a precursor to the antioxidant glutathione, protects cells from damage caused by ROS, which trigger oxidative stress that can lead to cell death [8]. We investigated whether N-acetylcysteine (NAC) could counteract the harmful effects of ROS generated by both pro-oxidant therapies. We used a non-toxic concentration of NAC (5 μM) before applying each therapy. NAC pretreatment significantly improved cell viability in all three GBM cell lines following pro-oxidant therapies (Figure 4). It reduced the cytotoxicity of various DOX concentrations in U87MG cells and in PDT treatment at 0.1 and 0.25 mM of Me-ALA. As expected, U87MG cells were more sensitive exhibiting higher production of oxidative stress post-treatment. This finding supports the idea that ROS-mediated cytotoxicity is a key mechanism in this cell line, and NAC effectively protects against it. 

However, the protective effect of NAC was limited in the other two cell lines (T98G and LN229). It only reversed the negative effects of higher DOX concentrations (1 and 5 µM for T98G and 5 μM for LN229), which also caused a significant increase in ROS levels (Figure 3A). In the case of Me-ALA-based PDT, NAC’s reversal effect was even weaker compared to DOX. This is likely because Me-ALA PDT generates much higher ROS levels, overwhelming the antioxidant capacity of the cells, including the effects of NAC.

To clarify the concepts and summarize the main results, Table 1 lists the key characteristics of the GBM cell lines (LN229, U87MG, and T98G) in response to the pro-oxidant therapies Doxorubicin (DOX) and Photodynamic Therapy (PDT).

### 2.4. Synergistic Pro-Oxidant Combination of PDT and DOX Treatments

A therapeutic combination study was conducted to identify potential synergistic effects between the evaluated drugs. By integrating a chemotherapy regimen that targets both tumor cells and their surrounding environment with the cytotoxic effects of selective PDT, this approach could serve as a supplementary therapeutic option to surgery. This combined treatment strategy was developed to address the current difficulties in treating GBM. The combination treatment involved exposing three GBM cell lines to varying concentrations of DOX, followed by the addition of varied amounts of Me-ALA in a radiation regimen similar to that used in monotherapy (Figures S6 and S7). The concentrations evaluated were based on previous monotherapy experiments and are near the IC50.

**Figure 5:**
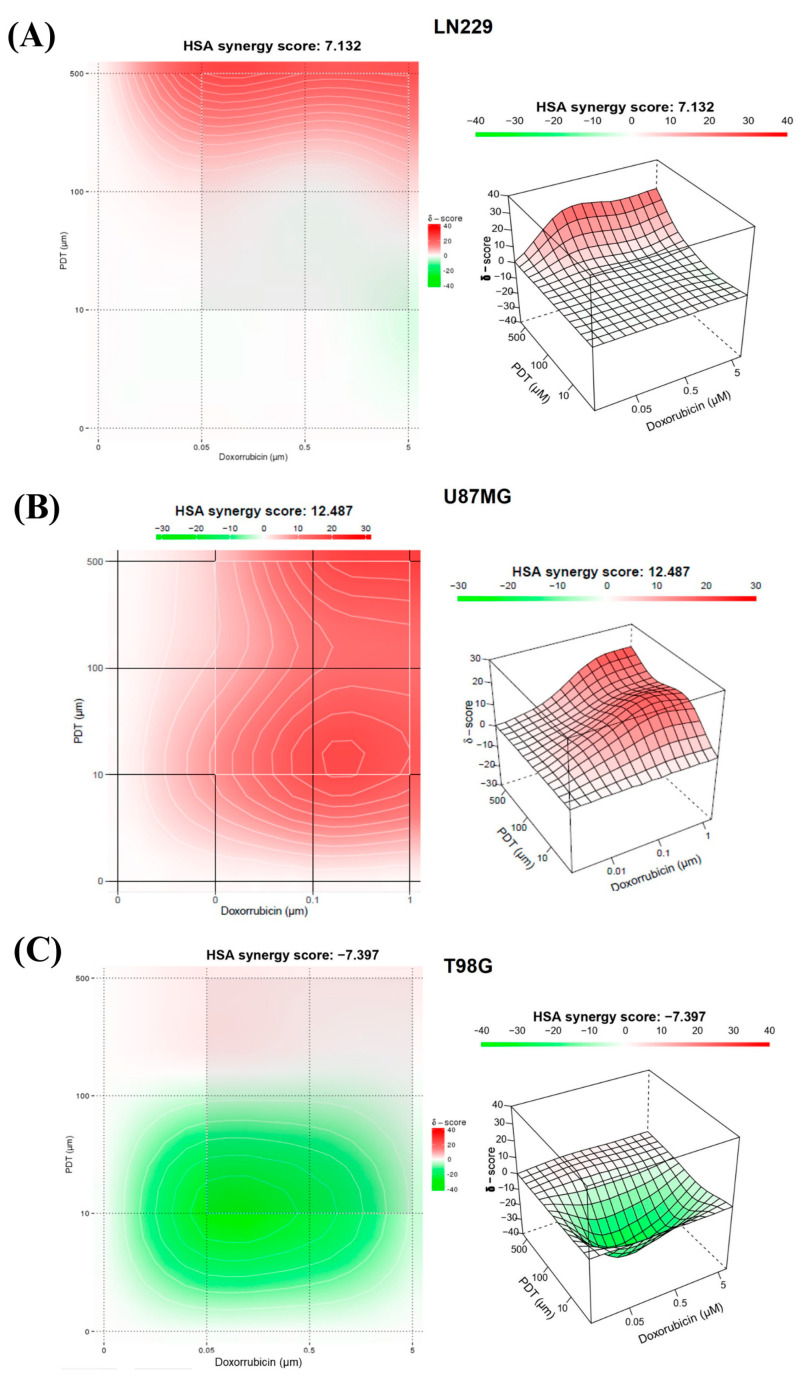
Evaluation of the synergistic combination of PDT and DOX treatments. The 3D images show synergy between the combination of DOX and PDT with Me-ALA based on HSA score for different GBM cell lines; LN229 (**A**), U87MG (**B**) and T98G (**C**). The graph was generated using a synergy finder, an online-based tool to determine synergy.

The combination of both treatments in LN229, as indicated by the matrix (Figure S5), showed that the highest percentages of inhibition were achieved with the highest concentrations of both Me-ALA and DOX (60–90%). In this sense, an additive effect in the combination of treatments for this cell line could be determined (Figure 5A), along with the possibility of therapeutic synergy for the combined treatment with the highest tested concentrations of 5 mM Me-ALA and 5 μM DOX. The synergy score for the LN229 cell line was 7.132. 

The therapeutic combination for the U87MG cell line generated greater cytotoxic effects than those observed for the monotherapies of DOX and PDT. Upon analyzing the combination matrix, it was observed that percentages of inhibition above 60% were obtained for more combinations of both treatments compared to the other cell lines. The synergy score demonstrated a synergistic effect of the therapeutic combination, reflected by a score of 12.5 (Figure 5B).

Finally, the cell inhibition percentages corresponding to the combination of PDT with DOX presented in the matrix (Figure 5C) suggest that for any combination of these two treatments using a concentration equal to or greater than 0.1 mM of Me-ALA, cell inhibition percentages greater than 88% are achieved. The same occurs for any combination with the highest concentration of DOX, for which over 95% cell inhibition was obtained. The synergy score was −7.4, indicating an additive effect. These results were reflected in the levels of oxidative stress achieved with both therapies in combination. 

**Figure 6:**
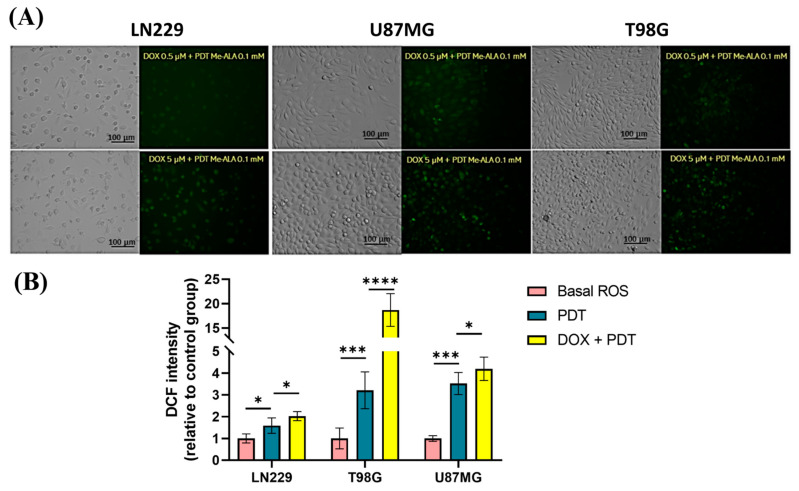
ROS production evaluation after combination therapy. (**A**) Fluorescence microscopy images of intracellular ROS levels evaluated immediately after combination treatments, scale bar = 100 µm. (**B**) Geometric mean fluorescence intensity quantification relative to autofluorescence of control group for LN229, U87MG and T98G after DOX and PDT treatments. ROS levels were determined immediately after PDT treatment with DCFDA assay using flow cytometry. Statistical analysis was performed using one-way ANOVA with Tukey’s post-hoc test. Significance levels are denoted by asterisks * *p* < 0.05, *** *p* < 0.001 and **** *p* < 0.0001.

A methodology was developed to evaluate the oxidative stress elicited by the treatment combinations in a synergistic modality. It included exposure to 0.5 and 5 μM DOX for 24 h, followed by incubation with 0.1 mM Me-ALA and irradiation of 1 J/cm^2^. Following the administration of both therapies, the degree of oxidative stress was assessed utilizing the DCF probe. Oxidative stress levels increased in all three cell lines when both combination treatments were used, as opposed to when PDT was used alone (Figure 6A). The studied combination exhibited significantly higher levels in the U87MG and T98G cell lines compared to lower values in the LN229 cell line (Figure 6B).

### 2.5. Reactive Oxidative Homeostasis Evaluation in GBM Cell Lines

**Figure 7:**
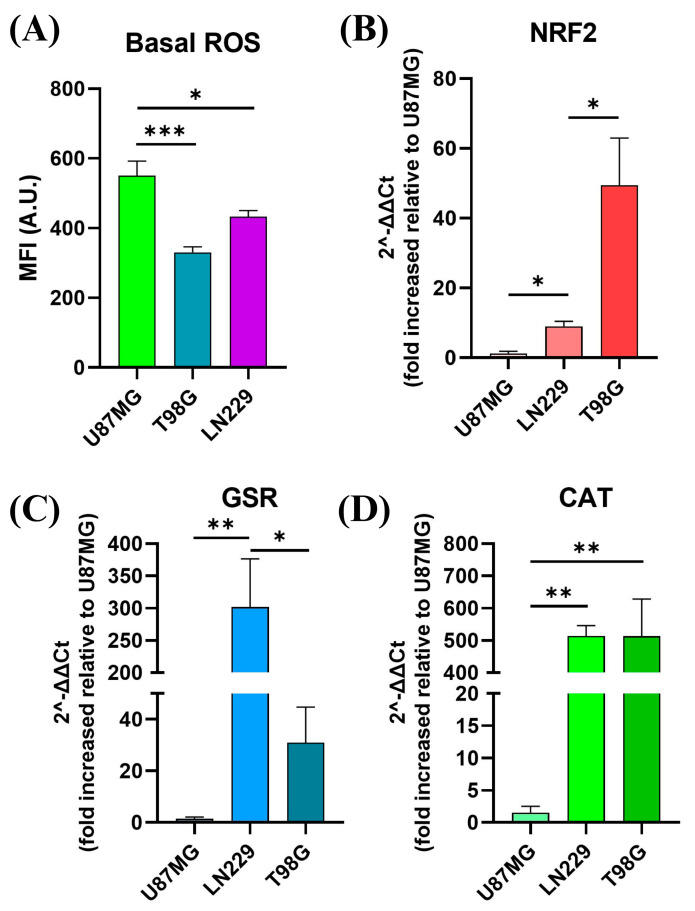
Basal redox homeostasis in GBM cell lines. (**A**) Geometric mean DCF fluorescence intensity quantification in GBM cell lines under basal growth conditions. Relative quantification of NRF2 (**B**), glutathione superoxide reductase (GSR) (**C**) and catalase (**D**) gene expression. Relative expressions across all cell lines were normalized by ACTB and relativized to the gene expression in U87MG. Statistical analysis was performed using one-way ANOVA with Tukey’s post-hoc test. Significance levels are denoted by asterisks * *p* < 0.05, ** *p* < 0.01 and *** *p* < 0.001.

GBM cancer cells exhibit a notable characteristic of having both high amounts of ROS and excessive production of intracellular antioxidant enzymes, which distinguishes them from normal cells [8,27,28]. In order to examine the function of basal oxidative stress homeostasis in GBM cells, we analyzed the basal ROS level and mRNA expression levels of the most pertinent antioxidant enzymes and related genes in the three GBM cell lines. The objective was to determine if these factors could be used as biomarkers to predict the effectiveness of ROS-based therapies. Basal ROS levels and the expression of genes related to the oxidative response were evaluated among GBM cell lines. Results are presented in Figure 7. Our results indicated significant heterogeneity in basal ROS levels across the cell lines, with U87MG exhibiting the highest ROS levels, followed by LN229 and T98G, which demonstrated intermediate and low ROS levels, respectively (Figure 7A). In parallel, the mRNA levels of key antioxidant response genes, including NFE2L2 (NRF2), glutathione superoxide reductase (GSR), and catalase (CAT), were quantitatively analyzed by the 2^−ΔΔCt^ method (Figure 7B–D). Notably, T98G and LN229 showed markedly elevated mRNA levels of these genes compared to U87MG, suggesting an improved compensatory upregulation in response to oxidative stress. Conversely, U87MG, with the highest ROS levels, exhibited the least expression of antioxidant response mRNA. These findings underscore the variability in oxidative stress management among GBM cell lines and highlight the potential for differential sensitivity to ROS-targeted therapies.

### 2.6. Comprehensive Analysis of Oxidative Stress-Related Genes in Glioblastoma and Their Relationship with TP53 and PTEN Mutations

**Figure 8:**
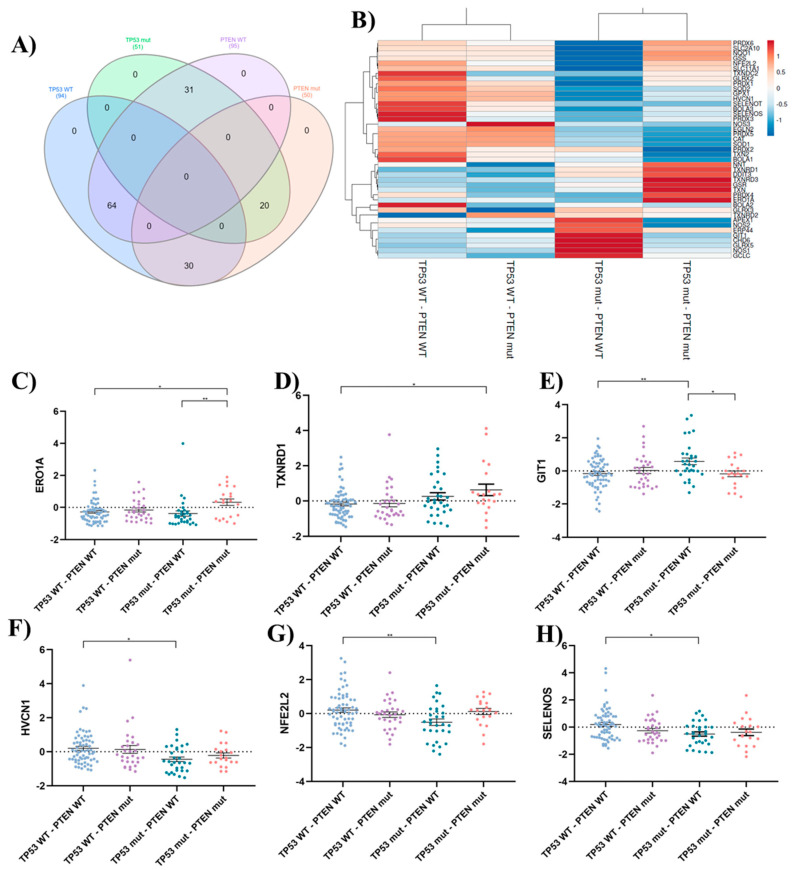
In silico analysis of oxidative stress-related genes in GBM. (**A**) Venn diagram, created using InteractiVenn, illustrates the distribution of patients across groups based on their *TP53* and *PTEN* mutation status. The overlapping areas depict the number of patients harboring mutations in both genes or only one of them. This figure provides a visual representation of the patient stratification used in the subsequent analyses. (**B**) The heatmap depicts the expression patterns of oxidative stress-related genes across GBM patient groups classified based on *TP53* and *PTEN* mutation status. The analysis was performed using ClustVis. The color intensity represents the relative expression level of each gene across samples. Red indicates high expression, while blue represents low expression. (**C**–**H**) Dot plots showing statistically significant differences in mRNA expression levels of oxidative stress-related genes across patient groups categorized based on *TP53* and *PTEN* mutation status. The groups include: TP53 WT—PTEN WT (*n* = 94 patients): Patients with wild-type (WT) TP53 and PTEN genes; TP53 WT—PTEN mut (*n* = 30 patients): Patients with WT TP53 and mutated (mut) PTEN genes; TP53 mut—PTEN WT (*n* = 31 patients): Patients with mutated TP53 and WT PTEN genes; TP53 mut—PTEN mut (*n* = 20 patients): Patients with mutated TP53 and PTEN genes. Statistical analysis was performed using one-way ANOVA with Tukey’s post-hoc test. Significance levels are denoted by asterisks: *p* < 0.001 (**) and *p* < 0.05 (*).

To investigate the role of oxidative stress in GBM, we conducted a comprehensive analysis of oxidative stress-related genes. The gene list was curated from the Gene Ontology (GO) term GO:0045454 (“cell redox homeostasis”) (https://amigo.geneontology.org/amigo/term/GO:0045454) (accessed 26 January 2024) and supplemented with relevant literature findings. An in silico gene expression analysis was conducted using the TCGA Pan-Cancer Atlas for GBM patient data obtained from cBioPortal. Considering the documented involvement of *PTEN* and *TP53* mutations in oxidative stress modulation [21,22,23,24,29,30,31], 145 GBM patients were stratified into four groups based on their mutation status (Figure 8A):

- *TP53* WT—*PTEN* WT (*n* = 64): Patients with wild-type (WT) *TP53* and *PTEN* genes.
- *TP53* WT—*PTEN* mut (*n* = 30): Patients with WT *TP53* and mutated (mut) *PTEN* genes.
- *TP53* mut—*PTEN* WT (*n* = 31): Patients with mutated *TP53* and WT *PTEN* genes.
- *TP53* mut—*PTEN* mut (*n* = 20): Patients with mutated *TP53* and *PTEN* genes.

To visualize relationships within the data, a heatmap was generated (Figure 8B). Rows were centered and scaled to ensure all features were on an equal footing. Hierarchical clustering was performed using correlation distance and average linkage for both rows and columns. This analysis identified two distinct clusters. Interestingly, the clustering primarily segregated samples based on the mutational status of the *TP53* gene. Group 1 contained samples with wild-type *TP53* regardless of *PTEN* mutation status, while Group 2 included samples with mutated *TP53*, again with *PTEN* mutation playing a secondary role in cluster assignment. 

Of the 43 genes analyzed, 6 exhibited statistically significant differences between some of the groups studied. Patients with double mutations (*TP53* mut and *PTEN* mut) showed upregulation of ERO1A (Figure 8C) and TXNRD1 (Figure 8D). ERO1A, encoded by the *Ero1a* gene, is crucial for protein folding but can also generate ROS as a byproduct, contributing to oxidative stress [32]. TXNRD1 is an oxidoreductase associated with elevated ROS levels and poor prognosis in cancer [33]. This pattern suggests a potential shift towards a pro-oxidant state in patients with combined *TP53* and *PTEN* mutations. This may explain the susceptibility of the T98G cell line (double mutant) to pro-oxidant antitumor therapies.

Interestingly, within *TP53* mutants, the presence of a *PTEN* mutation is linked to an upregulation of the pro-oxidant ERO1A and a downregulation of the antioxidant GIT1 (Figure 8E). GIT1 is known for its role in regulating the Keap1-Nrf2 signaling pathway, crucial for orchestrating the cellular antioxidant response [34]. In *PTEN* wild-type patients, overexpression of GIT1 was observed in the presence of *TP53* mutations. However, this heightened expression coincided with a decrease in the expression of key antioxidant genes such as *HVCN1* (Figure 8F), *NFE2L2* (Figure 8G), and Selenos (Figure 8H), indicating a compromise in the overall antioxidant defense system. Selenos, recognized for its antioxidant and anti-inflammatory functions, helps maintain endoplasmic reticulum (ER) morphology and regulates ER stress [35]. NRF2, a transcription factor encoded by the gene nfe2l2, acting as the master regulator of the cellular antioxidant response, binds to antioxidant response elements (ARE) within the promoter regions of numerous cytoprotective genes, including phase 2 detoxifying enzymes, thereby facilitating their expression and mitigating the effects of reactive electrophiles [36]. Additionally, recent insights suggest that the regulation of ROS production by VSOP/Hv1, a protein encoded by the hvcn1 gene, can be pro- or anti-oxidant, depending on age [37]. These findings could explain why the LN229 cell line exhibits the greatest resistance to pro-oxidant therapies. This cell line carries *PTEN* WT and *P53* mut P98L (C293T); the mutation on TP53, which does not affect its DNA binding ability, results in partial protein functionality—an effect that is not fully understood (functioning similarly to WT) [38]. This resistance would be further supported by its consistent overexpression of protective antioxidant systems.

These findings underscore the intricate interplay between *TP53* and *PTEN* mutations in modulating the oxidative stress response in GBM. *TP53* mutations seem to play a dominant role, potentially influencing the expression of both pro-oxidant and anti-oxidant genes. Further studies are needed to confirm these observations and explore the therapeutic potential of targeting specific components of the oxidative stress pathway in GBM based on *TP53* and *PTEN* mutation status.

## 3. Discussion

GBM cells utilize moderate amounts of ROS and reactive nitrogen species (RNS) to facilitate their proliferation and invasion [39]. It should be mentioned that certain mutations can lead to increased production of ROS and, for this reason, tumor cells consistently generate a greater amount of ROS compared to normal cells. However, the resulting oxidative stress can be counteracted by an increased overall antioxidant capacity, which is achieved through the buildup of antioxidant molecules as a result of an advanced adaptation [40]. GBM cells rely heavily on these systems to neutralize the harmful effects of ROS generation. Therefore, overwhelming the cells with excessive ROS production could be a potential strategy to eliminate tumor cells [8,41]. 

The impact of pro-oxidant therapies on GBM cell lines as monotherapies was assessed through a series of assays, shedding light on their effectiveness and underlying mechanisms. These cell lines are frequently employed in vitro models for GBM, exemplifying certain characteristics of the disease’s intrinsic intratumoral and intertumoral heterogeneity [42]. For instance, the T98G and LN229 cell lines harbor mutations in *TP53*, a gene frequently dysregulated across various tumor types and prevalent in both primary and secondary GBMs (30% and 65% incidence, respectively). In contrast, the U87MG cell line expresses the wild-type functional form of *TP53*. Cells with perturbations in the p53 pathway exhibit diverse capacities to mitigate oxidative stress and have been implicated in processes such as invasion, migration, proliferation, and evasion of apoptosis [43,44,45]. Notably, the M237I p53 mutation, found in the T98G cell line, has been associated with the acquisition of resistance to standard CTA [46,47]. It is evident that the basal oxidative stress varies among these cell lines, resulting in varied amounts of basal ROS (Figure 7A). These varying ROS levels are associated with distinct antioxidant responses. In this study, the antioxidant response was assessed by examining the gene expression of specific molecules involved in the antioxidant response. For instance, NRF2 is the primary transcription factor that controls the antioxidative response [36], and its expression levels differed among GBM cell lines (Figure 7B). Currently, many treatment approaches are being examined to inhibit this biological pathway [23,48]. 

DOX monotherapy revealed varying sensitivity among GBM cell lines (LN229, U87MG, T98G), with U87MG cells displaying the highest susceptibility and LN229 cells exhibiting the greatest resistance to DOX. These observations were supported by discernible cellular morphological changes indicative of apoptotic cell death. Conversely, PDT with Me-ALA exhibited divergent efficacy among the cell lines, with LN229 cells demonstrating notable resistance and U87MG and T98G cells displaying heightened susceptibility. PDT presents itself as a compelling approach to provoke cell death in tumor cells by generating ROS and thereby causing a redox imbalance. In addition, PDT utilizes light-activated PS to generate ROS, selectively inducing oxidative stress and apoptosis specifically in illuminated cancer cells, unlike DOX, which systemically generates ROS and affects both cancerous and healthy tissues. In this study, we opted to utilize the prodrug Me-ALA, which is a methylated variant that possesses greater lipophilicity compared to ALA. ALA is one of the primary PS examined in PDT for GBM [14,49]. Previously, we demonstrated greater resistance to PDT with PS based on conjugated polymer nanoparticles in T98G cells, which coincided with elevated expression levels of antioxidant enzymes and may be related to the mutational status of *TP53* [8]. In this study, PDT with Me-ALA induced effective cell death with an elevation of ROS surpassing the antioxidant levels possessed by the cell line, possibly due to the higher production of PpIX observed in these cells. Based on the outcomes of post-treatment cell viability assessments, including the generation of endogenous PS and cellular incorporation of DOX, it can be deduced that the LN229 cell line exhibits the greatest resistance to both treatments when used as monotherapies, despite having a higher level of cellular uptake.

Both DOX and PDT Me-ALA treatments led to increased ROS production in GBM cells, with higher ROS levels observed with PDT compared to DOX. U87MG cells exhibited the highest ROS levels post-treatment, indicating their heightened susceptibility to oxidative stress-induced cell death. Meanwhile, LN229, although it shares a mutation of the *TP53* gene, it is not the same mutation that the U87MG line has. Furthermore, the mutation status of the *PTEN* gene is different between both cell lines. This may suggest the differential behavior between both cell lines. Pre-treatment with NAC rescued cytotoxicity induced by DOX and PDT in U87MG cells, emphasizing the role of ROS in their cytotoxic mechanism. 

The GBM cell line LN229 exhibits various genetic and mutational characteristics that contribute to its aggressiveness and resistance to treatments, including chemotherapy and PDT. Numerous studies report that LN229 often shows amplification and activating mutations in the *EGFR* gene [50,51]. This can lead to constant activation of signaling pathways that promote cell growth and survival, even under adverse conditions such as chemotherapy, thereby increasing resistance to therapies. On the other hand, in LN229, the lack of mutation in IDH1 implies a different genetic profile and therapeutic response compared to gliomas with IDH1 mutations, where it has been observed that this gene mutation sensitizes tumor cells to various chemotherapeutic agents [52]. Other mutations of interest, which have a direct relationship with redox homeostasis and potential influence on pro-oxidant treatment, are analyzed below.

Various combination treatments involving PDT have demonstrated greater efficacy compared to treatments administered separately. For instance, one study evaluated the combined effect of PDT and TMZ for the treatment of GBM, revealing a synergistic effect and indicating its potential clinical use in glioma treatment [53]. In another study, the effects of combined therapy with PDT and a novel anti-angiogenic regimen using monoclonal antibodies against vascular endothelial growth factor receptors (VEGFR)-1 (MF1) and VEGFR-2 (DC101) were evaluated, demonstrating significant tumor growth inhibition and prolonged survival in animal models [54]. However, we have not found combined studies that evaluate the efficacy of pro-oxidant treatments in GBM, which makes our results encouraging for the potential application of such therapies.

Combining PDT with DOX resulted in synergistic cytotoxic effects in GBM cell lines, with U87MG cells showing the highest sensitivity to the combined treatment. Analysis of oxidative stress levels following combination therapy revealed increased stress levels across all cell lines, particularly pronounced in U87MG and T98G cells. The level of oxidative stress within cells reflects a balance between the rate of ROS production and the activity of detoxifying systems that neutralize them [55]. The increased basal oxidative stress in transformed cells, particularly in tumor cells harboring mutations in tumor suppressor genes such as *TP53* and *PTEN* throughout the carcinogenesis process, renders them highly dependent on their antioxidant systems to counteract the harmful effects of ROS [21]. Interestingly, this dependence could potentially be a vulnerability for cancer cells that have a mutated *TP53* gene. For instance, this vulnerability is demonstrated by the increased sensitivity of these cells to treatment with H_2_O_2_ [56]. However, previous research has also demonstrated that mutated p53 enhances the synthesis of antioxidant enzymes as a defensive reaction to the oxidative circumstances induced by the GOF activities of the mutant protein [22,43]. 

Furthermore, *PTEN* has a crucial role in the cellular metabolic regulation and response to oxidative stress. The main function of this is to protect cells from cell death caused by oxidative stress by regulating the PI3K/AKT signaling pathway to limit the production of oxidative stress [29,57]. However, when *PTEN* is lost, it disrupts the equilibrium of redox processes, leading to increased oxidative stress. This has the potential to affect the response of GBM cells to pro-oxidant treatments as well. A prior investigation has demonstrated that GBM U87MG cells harboring a mutant form of *PTEN*, which leads to its loss of function, display elevated levels of oxidative stress in comparison to those identical cells in which PTEN functionality has been reinstated by overexpressing the wild-type variant [29]. Therefore, we considered the mutational status of both *PTEN* and *TP53* in the three cell lines employed in this study. U87MG and T98G presented mutations in *PTEN* with loss of function of its enzymatic activity; meanwhile, LN229 had the wildtype *PTEN* gene.

Mutational analysis of *TP53* and *PTEN* genes revealed distinct gene expression profiles associated with the oxidative stress response. Patients with combined *TP53* and *PTEN* mutations showed upregulation of pro-oxidant genes like ERO1A and TXNRD1, suggesting a pro-oxidant shift in these cases. *TP53* mutations appeared to influence the expression of both pro-oxidant and antioxidant genes, with potential implications for treatment response. LN229 cells, characterized by *PTEN* wild-type and *TP53* mutant status, exhibited resistance to pro-oxidant therapies, potentially due to enhanced expression of protective antioxidant systems.

These findings highlight the complex interplay between pro-oxidant therapies, ROS production, and genetic factors in GBM, underscoring the importance of personalized treatment strategies targeting specific molecular pathways involved in oxidative stress response. Insufficient antioxidant defenses lead to oxidative stress, resulting in cell death when the harmful effects of ROS are not reduced. Further research is warranted to validate these observations and explore their clinical implications for GBM management.

## 4. Materials and Methods

### 4.1. Cell Lines and Culture Conditions

In this study, three distinct human GBM tumor cell lines were employed. The first cell line, U87MG (ATCC^®^ HTB-14™) with wild-type *TP53* and mutant *PTEN,* was originated from a GBM specimen obtained from an adult male patient. The second cell line, T98G (ATCC^®^ CRL-1690™), with mutated *TP53* (M237I) and *PTEN* genes, was derived from a GBM specimen obtained from a 61-year-old male patient. Finally, the third cell line, LN229 (ATCC^®^ CRL-2611™), was isolated from the right frontal parieto-occipital cortex of a 60-year-old Caucasian patient diagnosed with GBM. In contrast to the first two cell lines, this cell line harbors mutant *TP53* (P98L) and wild-type *PTEN*. All GBM cell lines were cultured in Dulbecco’s Modified Eagle’s Medium (DMEM, Sigma-Aldrich, St. Louis, MO, USA) supplemented with 10% fetal bovine serum (FBS, Internegocios, S.A, Buenos Aires, Argentina). In addition, a model of glial cells (specifically the B92 rat glial cell line) was employed to compare the primary outcomes. These cells were cultured in DMEM supplemented with 10% FBS.

### 4.2. Cell Uptake Analysis of DOX and Bioproduction of PpIX

GBM cell lines were cultured in 24-well plates with 20,000 cells per well and incubated at a temperature of 37 °C in a CO_2_ incubator for one night. Subsequently, the media was substituted with DOX (Glenmark Life Sciences, Mumbai, India) at different concentrations (0.1, 0.5, 1.5, and 10 μM). The cells were then incubated for 24 h, and the fluorescence of the CTA in the cells was assessed using flow cytometry in the red-B detector channel. On the other hand, to induce intracellular formation of PpIX, the three cell lines were seeded onto 24-well plates at a density of 20,000 cells. Subsequently, cells were exposed to media containing Me-ALA (Sigma) at concentrations of 0.01, 0.1, and 0.5 mM for a duration of 4 h. In order to quantify PpIX formation, cell fluorescence was subsequently analyzed by flow cytometry in the red-B channel.

Furthermore, GBM cell lines and glial B92 cells were placed on glass coverslips in 35 mm culture dishes at a density of 1.0 × 10^5^ cells/well in 24-well plates. They were then cultured overnight at 37 °C in 0.5 mL of media containing 10% FBS until they reached a confluency of 70–80%. Subsequently, the medium was extracted, and the cells were rinsed with PBS and exposed to DOX and Me-ALA at different concentrations. Following incubation, cells were subjected to three washes with PBS. Subsequently, cells were stained with MitoTracker^®^ Green FM (Invitrogen, Carlsbad, CA, USA) or Wheat Germ Agglutinin (WGA) Alexa Fluor 488-conjugated (Invitrogen, Carlsbad, CA, USA) for a duration of 20 min at room temperature. After staining, the cells were washed three times with PBS. Afterward, the nuclei were stained with Hoechst 33342 (Sigma) at a concentration of 1 µg/mL for a duration of 10 min. The coverslips were then placed over the slides and secured with Fluoromount^TM^ (Sigma) for subsequent evaluation using fluorescence microscopy.

### 4.3. Assessment of the Efficacy of DOX as a Single Treatment in GBM Cell Lines

The cell suspensions were prepared at a concentration of 200,000 cells per milliliter and seeded onto 96-well plates at a density of 20,000 cells per well. The cells were cultured for 24 h at a temperature of 37 °C in a CO_2_ incubator. Subsequently, various concentrations of DOX were examined within a range of 0.1–102.4 μM, with a duration of exposure of 24 h. After that time, the CTA medium was extracted from the plate. A solution of MTT in complete DMEM (0.5 mg/mL) was prepared, and 100 μL of this solution was added to each well. The plate was then incubated in darkness, under the same conditions as before, for a duration of 3 h. Next, the medium was disposed of and 100 μL of DMSO was introduced to dissolve the formazan crystals. Ultimately, the measurement of absorption was conducted utilizing a multiplate reader (Multiskan, Thermo Scientific, Waltham, MA, USA) at a specific wavelength of 570 nm. The absorption values of the treated groups were compared to the control group, which did not receive any treatment. The values were transformed into percentages, representing cell viability relative to the control, which was considered to have 100% viability.

### 4.4. Assessment of the Impact of TFD with Me-ALA on the Survival of GBM Cells

Cell viability was assessed following exposure to varying doses of Me-ALA in the three GBM cell lines. To achieve this, a 100 mM solution of the photosensitizer Me-ALA was prepared. Cell suspensions were prepared at a concentration of 200,000 cells per milliliter and seeded onto 96-well plates at a density of 20,000 cells per well. Afterward, solutions of Me-ALA were prepared in concentrations of 0.01, 0.1, 0.5, 1, and 2 mM in serum-free DMEM. The same medium was used for the control samples. The complete DMEM that was originally in the plates was removed, and two washes were performed with PBS 1×. The prepared Me-ALA solutions were then added to the plates, and the samples were incubated in the dark for 4 h. Following this period, irradiation was performed utilizing a multiLED system emitting light at a wavelength of 635 nm. The light dosage applied was 1 J/cm^2^, with a luminous output of 21.25 mW/cm^2^. Subsequently, the media was replaced with DMEM containing 10% SFB and the cells were incubated for 24 h. The following day, the feasibility of the cells was assessed using the MTT colorimetric test, as previously reported.

### 4.5. Reversal of Pro-Oxidant Cytotoxic Effects in Monotherapy with DOX and PDT through N-Acetylcysteine Intervention

The three GBM cell lines were seeded in 96-well plates at a density of 10,000 cells per well. On the next day, a solution containing N-acetylcysteine (NAC) in 10% FBS DMEM (5 µM) was introduced to the specified wells and left to incubate for 24 h. Following this period, the cells were subjected to DOX treatment at concentrations of 0.1, 1, and 5 µM for an additional 24 h. Certain wells were administered chemotherapy exclusively, without the inclusion of NAC. In addition, GBM cell lines were subjected to pre-incubation with Me-ALA (0.1–0.25–0.5 mM) for a duration of 24 h before being incubated with NAC as described previously. The cells were exposed to 1 J/cm^2^ of light after being incubated with Me-ALA for 4 h. Cell viability was assessed 24 h after treatments using MTT, and a comparison was made between wells that were incubated with NAC (NAC+) and those that were not (NAC−).

### 4.6. Cytotoxicity Effect of Combination of Doxorubicin and PDT with Me-ALA

We performed an experiment to evaluate the effect of combining DOX and PDT with Me-ALA on the viability of three different cell lines (U87MG, T98G, and LN229). Cells were placed at a concentration of 20,000 cells per well in 96-well plates for this experiment. After 24 h of culturing, the cells were treated with different concentrations of DOX close to the IC50 values (0.05, 0.5, and 5 µM for T98G and LN229, and 0.01, 0.1, and 1 µM for U87MG) for 24 h. After removing DOX, cells were treated with Me-ALA at concentrations of 0.01, 0.1, and 0.5 mM for 4 h, then subjected to irradiation of 1 J/cm^2^. Cell viability in each treatment was evaluated using an MTT assay after 24 h.

### 4.7. Determination of Synergy between Doxorubicin and PDT with Me-ALA

The interaction of DOX and PDT with Me-ALA was assessed using SynergyFinder, a tool available at https://synergyfinder.fimm.fi/ (accessed on 19 December 2023). The analysis was conducted in May 2024. SynergyFinder is a web-based application that assesses the synergy between a combination of medications using different reference models. In order to do this, we utilized the Highest Single Agent (HSA) model. This model measures the level of synergy by comparing it to the greatest response of a single agent. It suggests that the predicted combined impact is equal to the maximum response of a single drug at the corresponding concentrations. Summarized synergy scores represent the mean additional reaction resulting from pharmacological interactions. For example, a synergy score of 15 equates to a 15% response over the anticipated level. The acquired synergy scores were categorized as synergistic (synergy score > 10), additive (synergy score ranging from −10 to 10), or antagonistic (synergy score < −10).

### 4.8. Evaluation of Intracellular Oxidative Stress after Mono and Combo Treatment

GBM cell lines were seeded into 24-well plates with 500 μL of a prepared cell suspension containing 100,000 cells/mL per well, resulting in a total of 50,000 cells in each well. These plates were then incubated for 24 h at 37 °C with 5% CO^2^.

Following the incubation period, the medium was aspirated from all wells, and distinct concentrations of DOX (0.1, 0.5, 1.5 and 10 μM), approximating the IC50 value, were applied for 24 h. Additionally, three concentrations of Me-ALA near the IC50 (0.01, 0.1 and 0.5 mM) were administered for 4 h, followed by irradiation with a light dose of 1 J/cm^2^. Immediately after these separate treatments, the medium was aspirated, and a solution of CM-H2DCFDA in PBS at a concentration of 10 μM was introduced.

For the combined treatment, cells were seeded as previously described and incubated with DOX at 0.5 μM for 24 h, followed by incubation with Me-ALA at 0.1 mM, and irradiation with a light dose of 1 J/cm^2^. DCF fluorescence was assessed immediately after PDT treatment. To serve as a positive control, certain wells were treated with a solution of hydrogen peroxide (30% *v*/*v*, diluted 1/100 in PBS) and incubated for 20 min at 37 °C. Following a 30 min incubation period with the oxidative stress probe, the medium was aspirated, and the complete medium was added for a further incubation period of 10 min. Subsequently, the medium was removed, wells were rinsed with 1× PBS, and cells were detached using 1% trypsin.

The samples underwent analysis using flow cytometry (Guava easycyte 6 2L, Merck, Rahway, NJ, USA), with the green-B detector utilized to analyze the fluorescence of the DCF probe.

### 4.9. Gene Expression Analysis of Antioxidant Response in GBM Cell Lines

A quantitative polymerase chain reaction (qPCR) was conducted to assess the gene expression of several molecules involved in the antioxidant response of GBM cell lines. RNA was isolated from U87MG, LN229, and T98G cells cultured under growth conditions using TRIzol reagent (Invitrogen, Thermo Fisher Scientific, Carlsbad, CA, USA). The extracted RNA was then reverse transcribed using M-MLV reverse transcriptase (Invitrogen, Thermo Fisher Scientific) following the manufacturer’s instructions. The qPCR measurement was conducted using the SYBR Green qPCR Master Mix from Agilent Technologies, Santa Clara, CA, USA. Each reaction contained 10 ng of cDNA and was performed on Agilent’s Stratagene Mx3000PRO equipment. [58]. ACTB gene expression was used as a housekeeping gene to normalize the expression of target genes and the 2^−ΔΔCT^ method was used to calculate relative levels of gene expression using the Strata gene MxPro QPCR software v3.00 tool (Stratagene, Agilent Technologies). The quality of amplification was confirmed using a typical melting-curve cycle. The samples were subjected to triplicate analysis, and the experiment was replicated two times. Forward and reverse primers for the genes of interest are shown in Table 2.

### 4.10. Expression Analysis of Cell Redox Homeostasis Genes in GBM Patient Samples

In silico analysis leveraged the TCGA Pan-Cancer Atlas data accessible through cBioPortal (accessed May 2024) [59]. This analysis focused on 145 glioblastoma (GBM) patients. Mutation status of *TP53* and *PTEN* genes, along with mRNA expression of relevant genes, was retrieved for diploid samples. Venn diagrams were generated using InteractiVenn [60], while heatmaps were constructed using the online platform ClustVis [61]. Patients were stratified into four groups based on *TP53* and *PTEN* mutation status: TP53 WT—PTEN WT (*n* = 94): patients with wild-type (WT) TP53 and PTEN genes; TP53 WT—PTEN mut (*n* = 30): patients with WT TP53 and mutated (mut) PTEN genes; TP53 mut—PTEN WT (*n* = 31): patients with mutated TP53 and WT PTEN genes; TP53 mut—PTEN mut (*n* = 20): patients with mutated TP53 and PTEN genes. The analysis focused on mRNA expression of genes associated with the Gene Ontology (GO) term “cell redox homeostasis” (GO:0045454) (https://www.ebi.ac.uk/QuickGO/term/GO:0045454) (accessed 1 May 2024) and was further supplemented by relevant literature findings on oxidative stress.

### 4.11. Statistical Analysis

The data collected from the cell uptake, ROS production, and cytotoxicity experiments were analyzed using Graph Prism 8 software (accessed 19 December 2023). One-way ANOVA was used to determine the significant differences in cell viability for cells treated with a single treatment of DOX and PDT with Me-ALA. The fluorescence intensity of DOX uptake and PpIX bioproduction was measured for each treatment group and normalized to the autofluorescence of untreated cells. The acquired data were subsequently analyzed using Graph Prism 8 software. Two-way ANOVA was used to determine the significant difference in cell uptake and intracellular bioproduction of PpIX among various treatment groups considering different concentrations and the behavior of cell lines. The experiments were conducted with a sample size of *n* = 6 and repeated twice.

## Figures and Tables

**Table 1:** Summary of key results for GBM cell lines (LN229, U87MG, T98G) in response to pro-oxidant therapies. IC50 values for DOX and PDT indicate the concentration at which 50% inhibition of cell viability is observed. Morphological changes refer to cellular alterations observed post-treatment. ROS levels indicate the amount of reactive oxygen species produced. Cellular uptake of DOX and PpIX production after Me-ALA treatment are quantified. Resistance to pro-oxidant therapies is marked as + (low resistance), ++ (moderate resistance), +++ (high resistance). NAC reversal of cytotoxicity describes the effectiveness of NAC pretreatment in mitigating the cytotoxic effects, noted as significant or partial at various concentrations.

Cell Line	DOX IC50 (µM)	PDT IC50 (mM of Me-ALA)	Resistance to Pro-Oxidant Therapies	Morphological Changes with DOX	Morphological Changes with PDT	ROS Levels with DOX	ROS Levels with PDT	Cellular Uptake of DOX	PpIX Production with Me-ALA	NAC Reversal of Cytotoxicity
U87MG	0.14 ± 0.1	0.33 ± 0.05	+	More alterations	More alterations	Higher	Highest	High	Highest	Significant at low conc.
T98G	0.5 ± 0.15	0.3 ± 0.1	++	More alterations	More alterations	Lower	Higher	Intermediate	High	Partial at high conc.
LN229	6.88 ± 0.6	0.8 ± 0.2	+++	Fewer alterations	Fewer alterations	Higher	Lower	High	High	Partial at high conc.

**Table 2:** qPCR primers designed on Primer-BLAST and verified on BLAST-N (NCBI).

Gene	Forward 5′-3′	Reverse 3′-5′	Product Length (bp)	NM
*ACTB*	ATTGCCGACAGGATGCAGAA	GCTGATCCACATCTGCTGGAA	150	NM_001101.5
*GSR*	TGGCACTTGCGTGAATGTTG	CACATAGGCATCCCGCTTTTC	157	NM_001195102.3
*NFE2L2*	TCAGCGACGGAAAGAGTATGA	CCACTGGTTTCTGACTGGATGT	174	NM_006164.5
*CAT*	GGCGAGGCAGCTTGAGTTAA	CACCGCCTCGGCTTGTC	331	NM_001752.4

## Data Availability

The raw data supporting the conclusions of this article will be made available by the authors on request.

## Supplementary Materials

The following supporting information can be downloaded at: https://www.mdpi.com/article/10.3390/ijms25147525/s1.
