# Supplementary material for: Enhancing Therapeutic Approaches in Glioblastoma with Pro-Oxidant Treatments and Synergistic Combinations: In Vitro Experience of Doxorubicin and Photodynamic Therapy
Source: Int J Mol Sci. 2024 Jul 9;25(14):7525. doi: 10.3390/ijms25147525 (PMC11277534; doi:10.3390/ijms25147525)
Supplement: Supplementary file 1 [file ijms-25-07525-s001.zip › ijms-3060462-supplementary.pdf]

Enhancing Therapeutic Approaches in Glioblastoma with Prooxidant Treatments and Synergistic Combinations: The in vitro experience of Doxorubicin and Photodynamic therapy

Bruno A. Cesca [1], Matías D. Caverzan [2,3], María J. Lamberti [1,4] and Luis E. Ibarra [1,4,*]

This section includes:

SUPPORTING FIGURES

Figure S1. PDT protocol standardization.

Figure S2. Visualization of the colocalization of PpIX, nuclei, and cell membranes

Figure S3. ROS production in GBM cells after treatment with doxorubicin (DOX) and photodynamic therapy (PDT), determined using flow cytometry.

Figure S4. ROS generation was assessed using fluorescence microscopy after treating B92 glial cells with doxorubicin (DOX) and photodynamic therapy (PDT).

Figure S5. Dose−response matrix (inhibition) from DOX and PDT treatment in combination.

Figure S6. Alterations in morphology of LN229 cells following DOX and PDT combination treatments using varying doses of DOX and Me-ALA and a light dose of 1 J/cm^2.

Figure S7. Alterations in morphology of U87MG cells following DOX and PDT combination treatments using varying doses of DOX and Me-ALA and a light dose of 1 J/cm^2.

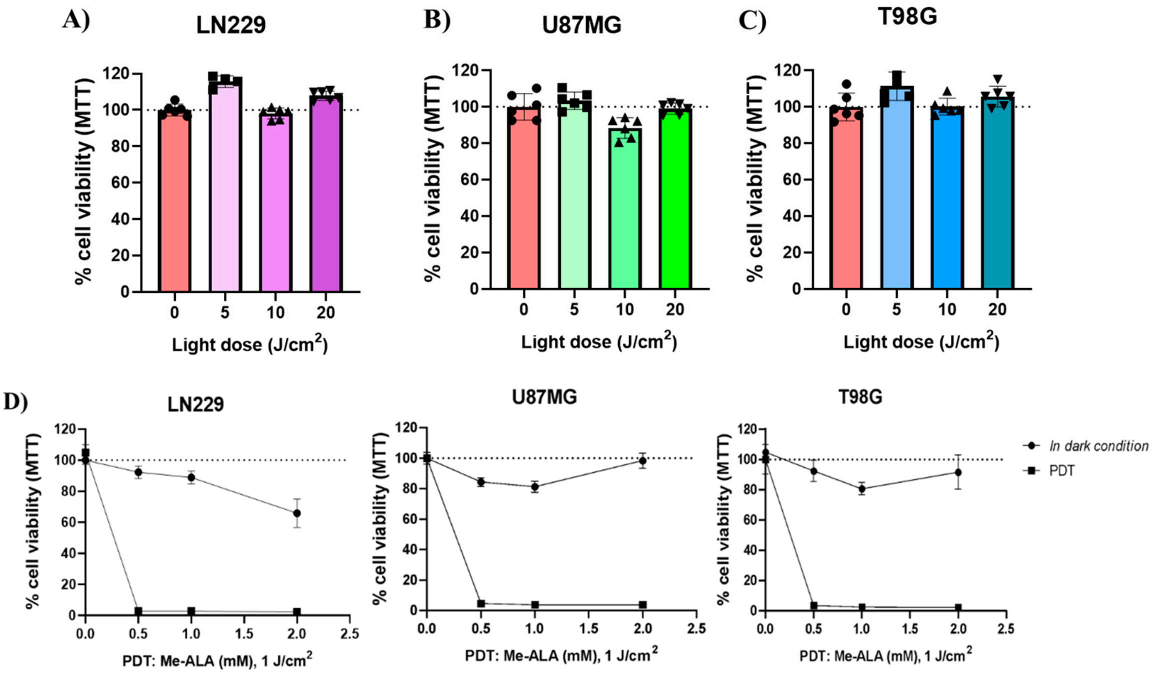

Figure S1. PDT protocol standardization. Assessment the cytotoxicity of light dosage alone in LN229 (A), U87MG (B), and T98G (C) cell lines. D) Assessment the cytotoxicity of Me-ALA concentration in dark condition in three GBM cell lines.

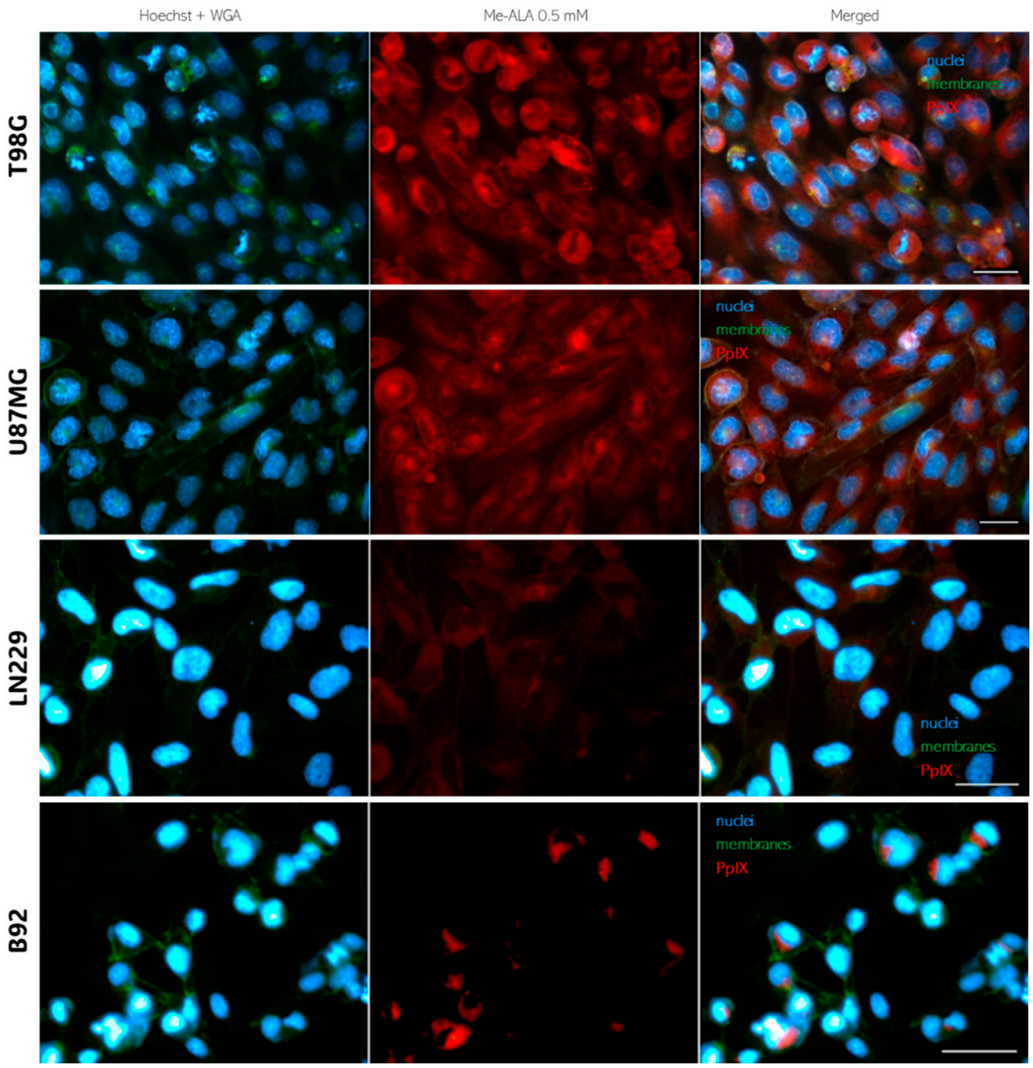

Figure S2. Fluorescence microscopy images of GBM and glial cells. Nuclei are stained with Hoechst 33342 (blue), cell membranes are stained with WGA (green), and PpIX fluorescence is shown in red. Colocalization analysis reveals the distribution and overlap of these markers within the cells. Scale bar: 50 µm.

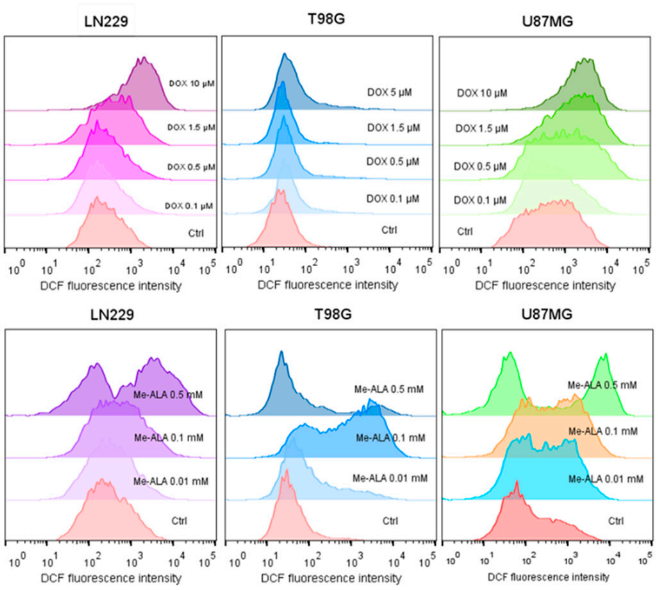

Figure S3. ROS production following doxorubicin (DOX) and photodynamic therapy (PDT) treatments. The upper panel displays histograms derived from flow cytometry analysis of DCF fluorescence intensity following treatment with various doses of DOX. The lower panel shows histograms acquired after photodynamic therapy (PDT) using different concentrations of Me-ALA and irradiation with a light dose of 1 J/cm^2.

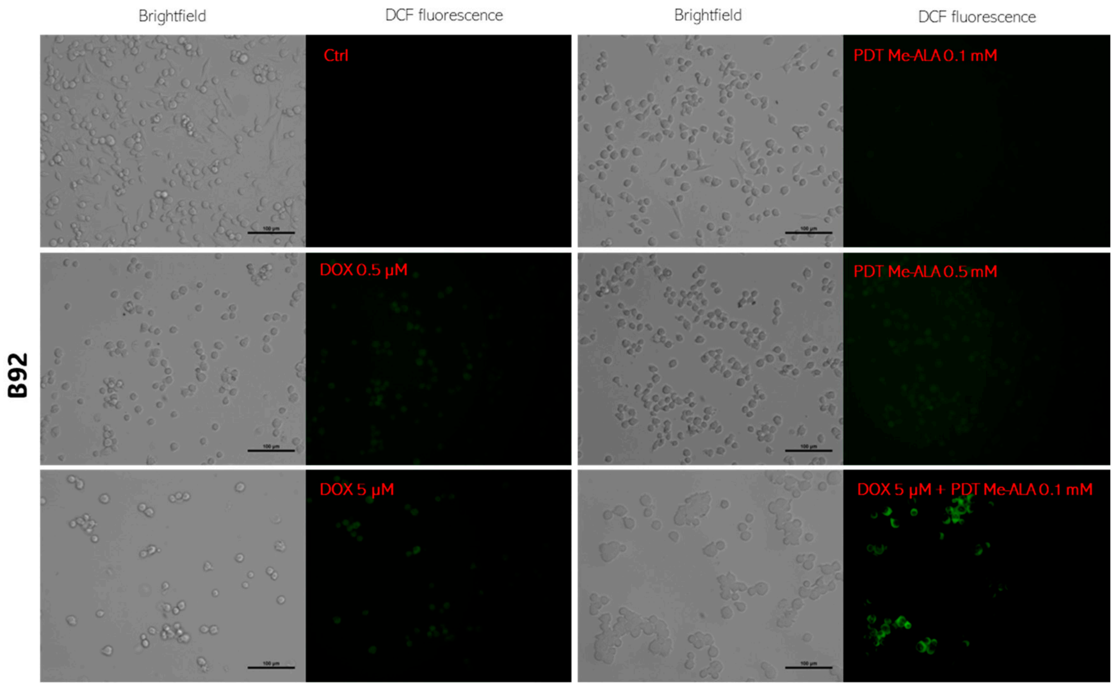

Figure S4. ROS production in B92 cells following doxorubicin (DOX) and photodynamic therapy (PDT) treatments and the combination. DCF fluorescence was capture immediately after treatments. Scale bar = 100 µm.

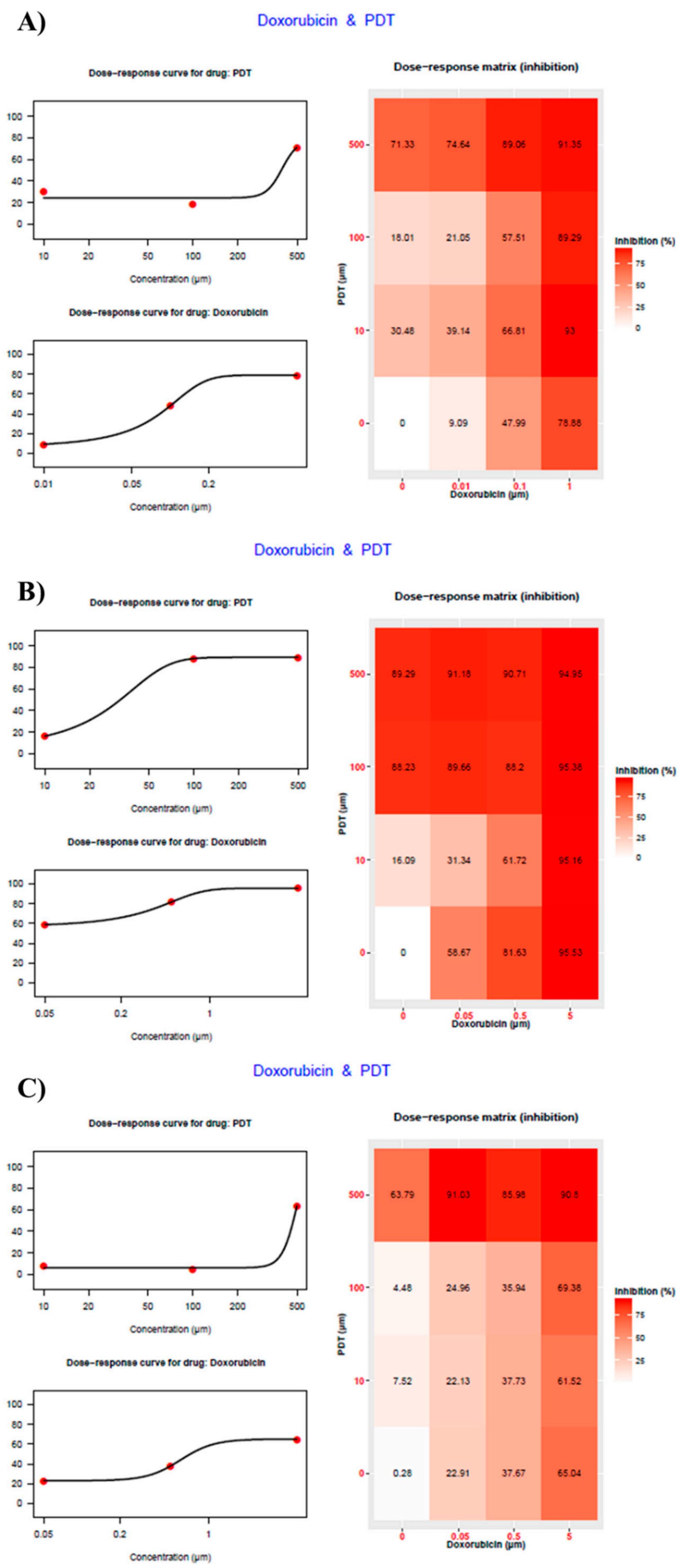
A)
Doxorubicin & PDT
Dose−response curve for drug: PDT
Concentration (μm)
Dose−response curve for drug: Doxorubicin
Concentration (μm)
Dose−response matrix (inhibition)
71.33 74.64 89.06 91.35
18.01 21.05 57.51 89.29
30.48 39.14 66.81 93
0 9.09 47.99 78.88
PDT (μm)
Doxorubicin (μm)
Inhibition (%)
B)
Doxorubicin & PDT
Dose−response curve for drug: PDT
Concentration (μm)
Dose−response curve for drug: Doxorubicin
Concentration (μm)
Dose−response matrix (inhibition)
89.29 91.18 90.71 94.95
88.23 89.66 88.2 95.38
16.09 31.34 61.72 95.16
0 58.67 81.63 95.53
PDT (μm)
Doxorubicin (μm)
Inhibition (%)
C)
Doxorubicin & PDT
Dose−response curve for drug: PDT
Concentration (μm)
Dose−response curve for drug: Doxorubicin
Concentration (μm)
Dose−response matrix (inhibition)
63.79 91.03 85.98 90.8
4.48 24.96 35.94 69.38
7.52 22.13 37.73 61.52
0.28 22.91 37.67 65.04
PDT (μm)
Doxorubicin (μm)
Inhibition (%)

Figure S5. Dose−response matrix (inhibition) from DOX and PDT treatment in combination. The synergy scores for drug combinations obtained for U87MG (A), T98G (B), and LN229 (C) were calculated and visualized using the synergy finder software.

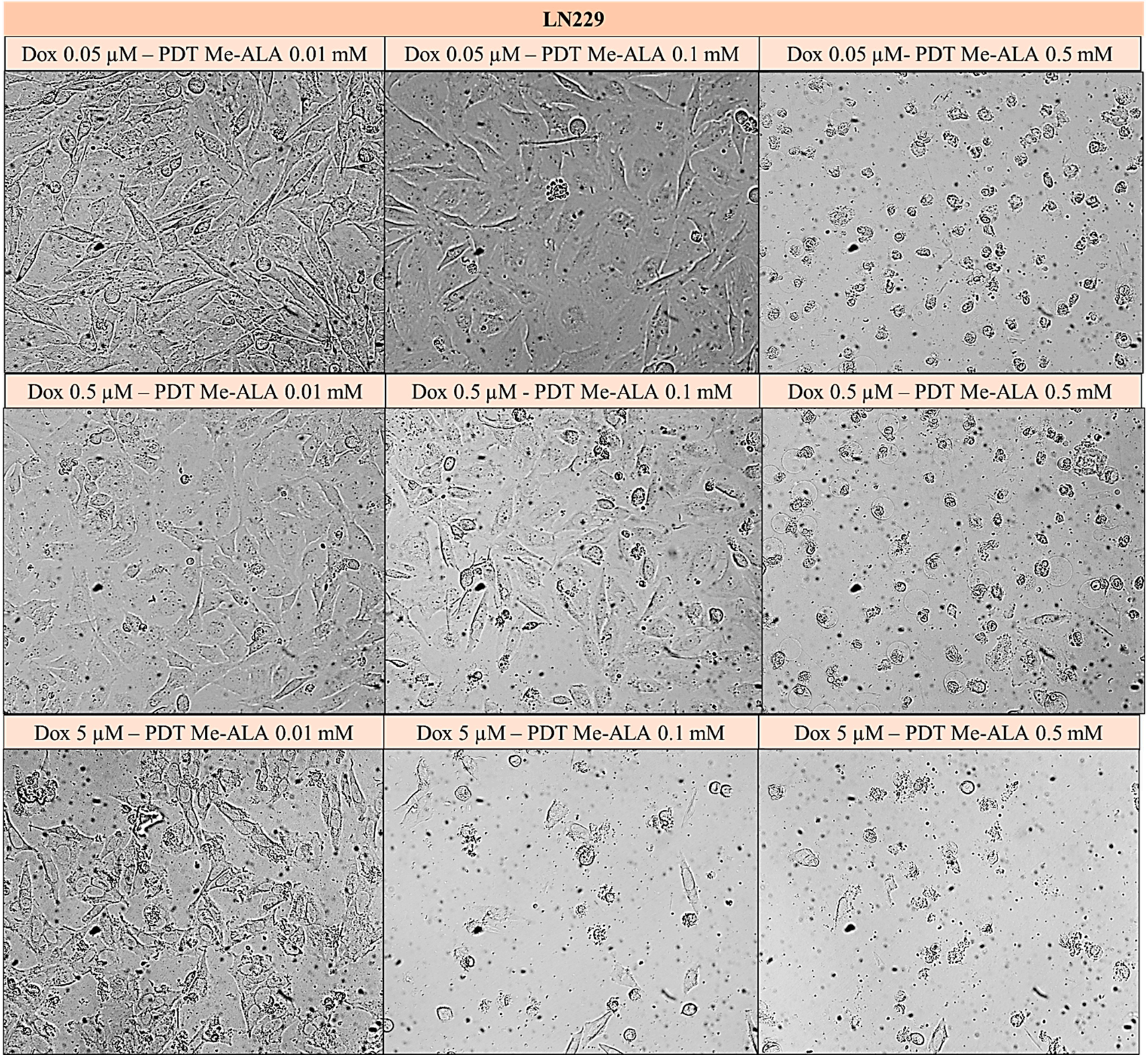

Figure S6. Alterations in morphology of LN229 cells following DOX and PDT combination treatments using varying doses of DOX and Me-ALA and a light dose of 1 J/cm^2. The cell lines were seen using bright-field imaging on an inverted optical microscope after being treated with DOX at concentrations of 0.05, 0.5 and 5 µM for 24 hours, followed by exposure to Me-ALA at concentrations of 0.01, 0.1, and 0.5 mM for a duration of 4 hours. Subsequently, the cells were subjected to irradiation using a red LED panel with a wavelength of 635 nm and an energy density of 1 J/cm^2. Magnification of 200x.

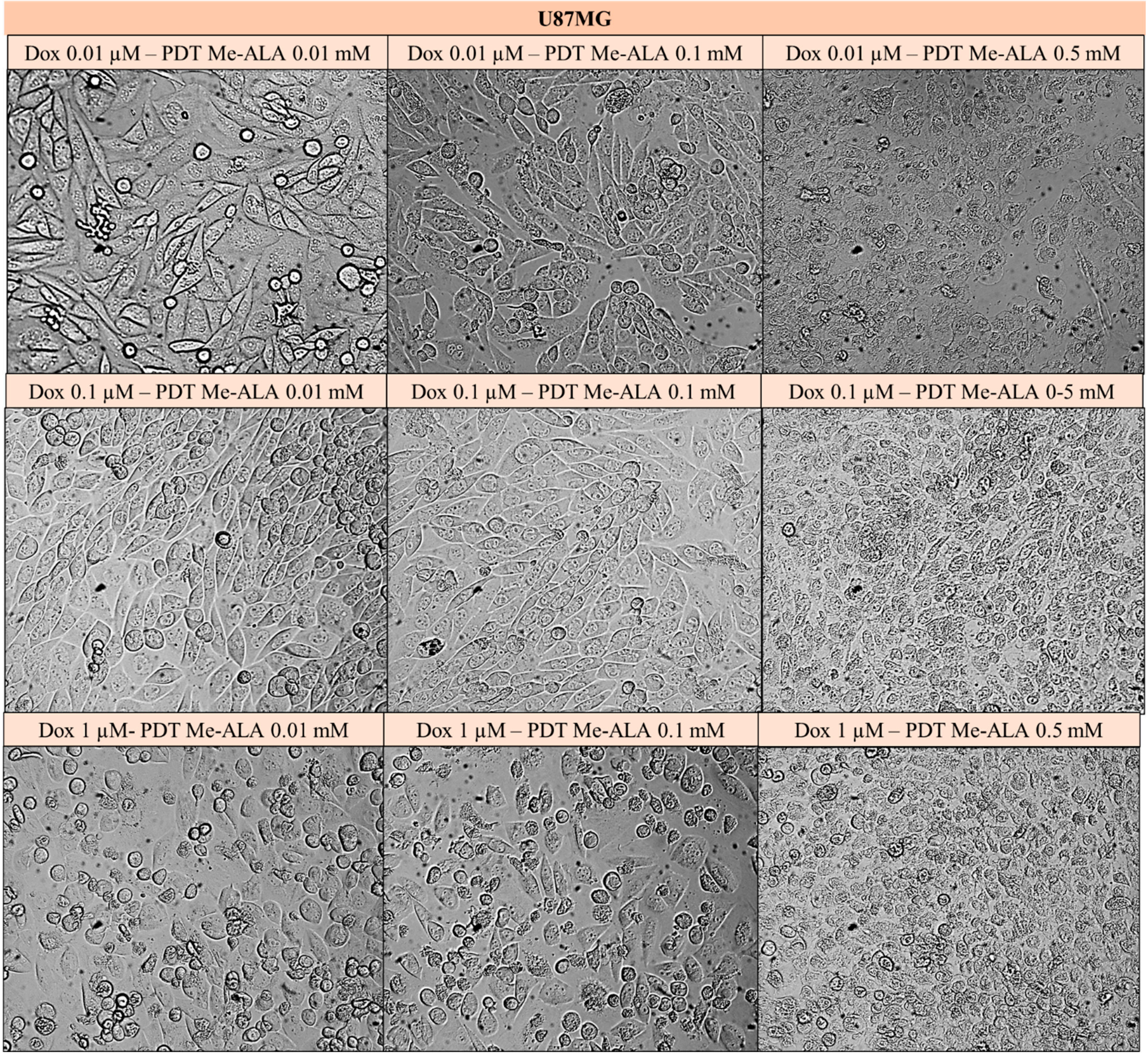

Figure S7. Alterations in morphology of U87MG cells following DOX and PDT combination treatments using varying doses of DOX and Me-ALA and a light dose of 1 J/cm^2. The cell lines were seen using bright-field imaging on an inverted optical microscope after being treated with DOX at concentrations of 0.01, 0.1 and 1 µM for 24 hours, followed by exposure to Me-ALA at concentrations of 0.01, 0.1, and 0.5 mM for a duration of 4 hours. Subsequently, the cells were subjected to irradiation using a red LED panel with a wavelength of 635 nm and an energy density of 1 J/cm^2. Magnification of 200x
